# Development of pGEMINI, a Plant Gateway Destination Vector Allowing the Simultaneous Integration of Two cDNA via a Single LR-Clonase Reaction

**DOI:** 10.3390/plants6040055

**Published:** 2017-11-12

**Authors:** Marino Exposito-Rodriguez, Philippe P. Laissue, Patricia E. López-Calcagno, Philip M. Mullineaux, Christine A. Raines, Andrew J. Simkin

**Affiliations:** 1 School of Biological Sciences, Wivenhoe Park, University of Essex, Colchester CO4 3SQ, UK; mexpos@essex.ac.uk (M.E.-R.); plaissue@essex.ac.uk (P.P.L.); pelope@essex.ac.uk (P.E.L.-C.); mullin@essex.ac.uk (P.M.M.); rainc@essex.ac.uk (C.A.R.); 2 Genetics, Genomics and Breeding, NIAB EMR, New Road, East Malling, Kent ME19 6BJ, UK

**Keywords:** gateway, gene stacking, LR clonase, pGEMINI, fluorescence

## Abstract

Gateway technology has been used to facilitate the generation of a large number of constructs for the modification of plants for research purposes. However, many of the currently available vectors only allow the integration of a single cDNA of interest into an expression clone. The ability to over-express multiple genes in combination is essential for the study of plant development where several transcripts have a role to play in one or more metabolic processes. The tools to carry out such studies are limited, and in many cases rely on the incorporation of cDNA into expression systems via conventional cloning, which can be both time consuming and laborious. To our knowledge, this study reports on the first development of a vector allowing the simultaneous integration of two independent cDNAs via a single LR-clonase reaction. This vector “*pGEMINI*” represents a powerful molecular tool offering the ability to study the role of multi-cDNA constructs on plant development, and opens up the process of gene stacking and the study of gene combinations through transient or stable transformation procedures.

## 1. Introduction

Gateway technology has become the cornerstone in the facilitation of the generation of a large number of constructs for the modification of plants for research purposes, and due to this, a wide array of gateway-adapted plant expression vectors have been developed over the last few years [1,2,3,4,5,6]. Modern studies of metabolic processes, such as photosynthesis, focus on the modification of multiple enzymatic steps in a given pathway to modify metabolic flux or yields. In the case of photosynthesis, multigene manipulation is at the forefront of maintaining food security [7,8].

Gene stacking defines the over-expression of multiple transcripts in a metabolic pathway, thereby increasing/modifying metabolic flux with the goal of improving/modifying the metabolic outcome [9]. Until recently, the initial goal of gene stacking has been accomplished in two ways: (1) Crossing lines carrying independent transgenes and the identification of plants carrying both transcripts of interest; and (2) Retransformation of plants already carrying a transgene. This can be accomplished using available vectors with different selection criteria (e.g., antibiotics). In more recent years, a third option has presented itself: Transformation of plants with two or more genes of interest integrated into a single construct. This system has the advantage of allowing the integration of two or more genes through a unique transformation event. To accomplish this task, a number of vectors have been developed, allowing for the parallel integration of several transcripts of interest into a single expression construct [10]. Most of these vectors, however, employ, in part, standard cut-and-paste methodology, which can be time consuming and laborious [10,11]. More recently, GoldenGate technology permits the generation of large multigene constructs for plant transformation [12,13]. However, this technology has the drawback of often requiring the generation of synthetic cDNA, promoters, and terminators at considerable expense.

Here, we report on the construction of the first vector permitting the dual-integration of two cDNA of interest via a single LR-clonase reaction. Finally we tested the successful LR incorporation of two independent cDNA into the pGEMINI system using a fluorescent protein and transient transformation approach.

## 2. Methods and Materials

### 2.1. Generation of pGEMINI Constitutive Double Expression Vector

**Figure 1:**
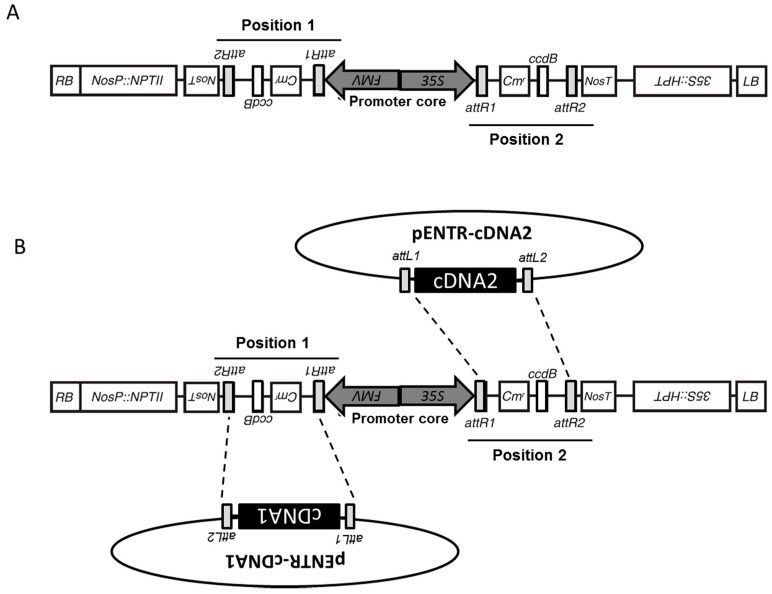
Schematic representation of vector pGEMINI. (**A**) Each of the two positions is represented and the promoter core shows the location of the back-to-back FMV and 35S promoters; (**B**) Schematic representation of LR clonase reaction showing the integration of two independent cDNAs into each clonase cassette.

The backbone of the pGEMINI vector was constructed in pGWB2 that had been previously generated from a modified pBI vector containing the hygromycin B phosphotransferase (*hpt*) and the Neomycin phosphotransferase II (*nptI*)*I* genes [2] (AB289765); thus, pGEMINI carries both hygromycin- and kanamycin-resistant cassettes (see Figure 1A). pGWB2 [2] (AB289764) was cut with *Hind*III (AAGCTT) overnight at 37 °C and treated with alkaline phosphatase (fermentas). The full-length LR site from the pDESTOE gateway vector [14], including promoter FMV [15,16] and the nos terminator, were amplified by PCR using primers pDESTF (5′CTGAGA-HindIII-TGAAGGCGGGAAACGACAATCTGATCC′3) and pDESTR (5′CTGAGA-HindIII-AGGCCTTCATAACGTGACTCCCTTAATTCTCC′3). The amplified product was digested with *Hind*III (Promega) and cloned into pGWB2 (Figure 1) in an inverse orientation to make vector pGEMINI. (Figure 1). *Position 1* is under the constitutive control of the FMV promoter and *Position 2* is under the constitutive control of the 35S promoter followed by the *nos* 3′ terminator. pGEMINI carries flanking resistance cassettes for kanamycin and hygromycin (see Figure 1).

### 2.2. Generation of pGEM-RED/YELLOW Expression Clones

The full-length cDNAs for red fluorescent protein (mRFP) was amplified using pGWB554 (AB294509) as template vector [6], and using primers clo_mRFP_F (5′caccATGGCCTCCTCCGAGGACGT′3) and clo_mRFP_R (5′TTAGGCGCCGGTGGAGTGG′3); the circularly permuted yellow fluorescent protein (cpYFP) was amplified from the vector Gateway HyPer-AS entry clone (Evrogen cat No. FP943) using primers clo_cpYFP_F (5′caccATGTCCGCCGGTTACAA′3) and clo_cpYFP_R (5′TCAGGTTCCGTTGTACTCTAG′3). mRFP and cpYFP were cloned into pENTR/D-TOPO (Invitrogen) to generate pENTR-mRFP (678 bp –cDNA1) and pENTR-cpYFP (759 bp –cDNA2), respectively. The full-length cDNAs for mRFP and cpYFP were cloned into the pGEMINI destination gateway vector by co-recombination with the pENTR-mRFP and pENTR-cpYFP entry clones (Figure 1B).

### 2.3. Plant Cultivation and Agrobacterium-Mediated Transient Transformation of Nicotina Benthamiana

pGEMINI recombined vectors pGEM-(Red/Red), pGEM-(Yellow/Yellow) and pGEM-(Yellow/Red) were electroporated into *A. tumefaciens* strain GV3101. The *A. tumefaciens* strains were grown overnight at 28 °C in LB medium supplemented with 50 mg/L kanamycin, 25 mg/L hygromycin, 250 mg/L Rifampicin. Cells were harvested by centrifugation (4500 rpm, 4 °C), resuspended in K-MES buffer (10 mM 4-Morpholineethanesulfonic acid sodium salt; 10 mM MgCl_2_, pH 5.6) and 150 mM acetosyringone, and diluted to an optical density (OD600 nm) of 0.6. Cultures were allowed to incubate for 4 h at room temperature in K-MES prior to infiltration. The suspension of each *Agrobacterium* for each vector to be tested was mixed in a 1:1 ratio with an *Agrobacterium* suspension carrying a vector pBIN19-p19 containing p19, a viral suppressor of gene silencing. Two leaves from 4- to 5-week-old wild-type *N. benthamiana* plants were infiltrated using a needleless 1 mL syringe. The plants were allowed to develop in a plant growth room (12 h light, 12 h dark). The infiltrated leaves were analyzed for GFP and mRFP fluorescence by confocal microscopy 3 days after infiltration.

For image acquisition, a Carl Zeiss LSM 510 META confocal microscope was used with a M27 20× plan-apochromatic lens with numerical aperture (N.A.) 0.8. Images were acquired in three channels, using one-way sequential line scans. cpYFP was excited with the 488 nm line of a 30 mW argon laser, 6.1 A, 15% transmission intensity, and its emission collected at 505–550 nm. mRFP was excited at 543 nm line of a 1 mW HeNe laser at 17.7% transmission intensity and its emission collected at 560–615 nm. Image size was 1024 × 1024 pixels, with a resolution of 6.77 pixels per μm. No offset was used, and the scan speed was ¼ frames/s (galvano scanner). Pinhole size was set to 1.2 times the Airy disk size of the 1.4 N.A. objective at 525 nm.

## 3. Results

### 3.1. Structure of pGEMINI

The backbone of the pGEMINI vector was constructed in pGWB2 that had previously been generated from a modified pBI vector containing *hpt* and *nptII* genes [2]; thus, pGEMINI carries both kanamycin- and hygromycin-resistant cassettes (see Figure 1A).

The full-length LR-site from the pDESTOE gateway vector [14], including the figwart mosaic virus promoter (FMV [15]) and the nopaline synthase gene (NOS) terminator, were amplified by PCR and cloned into the HindIII site of vector pGWB2 [2]. pGEMINI was selected for the integration of the second cassette in an anti-parallel orientation with the two promoters in back-to-back orientation forming a promoter core (see Figure 1A). The resulting 5626 bp antiparallel double LR-clonase-cassette (nos-attR2-CmR-ccdB-attR1-FMV-35S-attR1-ccdB-attR2-nos) contains a ccdB coding sequence and a chloramphenicol resistance gene in both clonase positions flanked by attR sites. Since the ccdB protein interferes with the *E. coli* DNA gyrase, inhibiting growth of most strains, pGEMINI must be propagated in *E. coli* strains resistant to ccdB toxicity (e.g., DB3.1 containing the gyrA462 allele, which renders the strain resistant to the toxic effects of the ccdB gene). In this manner, ccdB can be used to ensure double integration of the chosen cDNAs when propagated in ccdB-sensitive strains of *E. coli* (e.g., One Shot^®^ TOP10 and OmniMAX™). As shown in Figure 1, *Position 1* is under the constitutive control of the FMV promoter and *Position 2* is under the constitutive control of the 35S promoter.

### 3.2. Integration of cDNA from pENTR/D via an LR Clonase Reaction

The 5626 bp antiparallel double LR-clonase cassette facilitates the introduction of two independent cDNAs into the vector in a single Gateway attL x attR reaction (Figure 1B). It should be noted that the integration of cDNA1 and cDNA2 is random, resulting in a population of plasmids where each cDNA is integrated into either or both LR-clonase sites (e.g., cDNA1 + cDNA1; cDNA1 + cDNA2; cDNA2 + cDNA1; cDNA2 + cDNA2). The full-length cDNAs for mRFP and cpYFP were cloned into the pGEMINI destination gateway vector by co-recombination with the pENTR-mRFP (678 bp) and pENTR-cpYFP (759 bp) entry clones (Figure 1B). Recombinant pGEMINIs were selected on media containing 25 μg/mL hygromycin and 50 μg/mL kanamycin, as previously described for the pGWB backbone [2]. After culturing, colonies were screened by multiplex PCR for dual-integration of each cDNA into the corresponding clonase sites. In 15% of selected reaction clones, dual-integration, resulting in vector pGEMINI-(mRFP/cpYFP) or pGEMINI-(cpYFP/mRFP), was confirmed. Several negative clones (from the 85% showing no hetero-integration) were selected where it was demonstrated that homo-integration had occurred—pGEMINI-(mRFP/mRFP) or pGEMINI-(cpYFP/cpYFP)—and used as controls for further studies. Additional reactions using several cDNA of interest were used to determine the likelihood of successful dual-integration. Under the same experimental conditions used previously, selected cDNA (1182 bp + 690 bp) and (1123 bp + 570 bp) resulted in 25% and 30% of dual-integration respectively (data not shown).

Three constructs were identified following the LR-clonase reaction and used for transient transformation in *N. benthamiana* (pGEM-(cpYFP/mRFP), pGEM-(cpYFP/cpYFP) and pGEM-(mRFP/mRFP)) using *Agrobacterium* strain GV3101.

### 3.3. Agrobacterium-Mediated Transient Transformation of Nicotiana Benthamiana and Fluorescent Protein Imaging

*N. benthamiana* wild-type plants were grown in a controlled environment at 22 °C with a 12-h photoperiod and 75% relative humidity. The *Agrobacterium* suspension for each construct to be tested was mixed in a 1:1 ratio with an *Agrobacterium* suspension carrying a vector pBIN19-p19 containing a viral suppressor of gene silencing. The infiltrated leaves were analyzed for mRFP and cpYPF fluorescence by confocal microscope five days after infiltration. The expression of the FPs was analyzed using laser-scanning confocal microscopy, and pGEMINI-(cpYFP/cpYFP) and pGEMINI-(mRFP/mRFP) were used as a control to determine if there was any crosstalk/bleed-through of the cpYFP signal into the mRFP channel, and vice versa.

**Figure 2:**
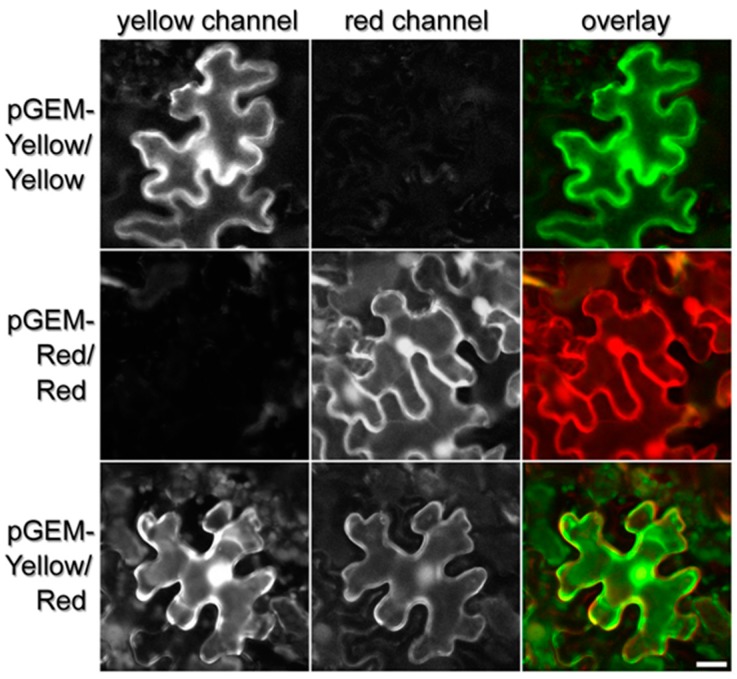
Confocal sections of the lower epidermis of *N. benthamiana* leaves, 5 days post-inoculation. Rows show the different pGEMINI constructs as indicated, while columns show images acquired in the yellow (**left**) and red channel (**right**). The scale bar represents 20 μm. Image size was 1024 × 1024 pixels, with a resolution of 6.77 pixels per μm. No offset was used, and the scan speed was ¼ frames/s (galvano scanner). Pinhole size was set to 1.2 times the Airy disk size of the 1.4 N.A. objective at 525 nm. Scanner zoom (3.0) was centered on the optical axis to minimize aberrations.

To identify yellow fluorescence, tissue samples were excited at 488 nm, and their emissions collected through a 505–550 nm bandpass filter. Transient expression of plasmid pGEMINI-(cpYFP/cpYFP) resulted in an accumulation of the yellow fluorescent protein in the cytosol (Figure 2; top row). Illuminating the same sample at 543 nm, the wavelength for the excitation of mRFP, only unspecific emissions were collected at 560–615 nm. This shows that there is minimal bleed-through between the two fluorescent proteins cpYFP and mRFP. Concomitantly, we used pGEMINI-(mRFP/mRFP) to analyze the fluorescence of the red and yellow spectra. Transient expression of plasmid pGEMINI-(mRFP/mRFP) resulted in no yellow fluorescent emissions following excitation (Figure 2; right row). When this material was analyzed by excitation for mRFP, a clear emission was collected, indicating that the mRFP is expressed with no bleed-through or cross-talk of the yellow spectra.

The expression of pGEMINI-(cpYFP/mRFP) resulted in the accumulation of cpYFP (Figure 2; bottom row) and mRFP in the cytosol of transformed plants (Figure 2; bottom row). These results demonstrate that pGEMINI permits the integration and expression of two cDNAs of interest in plant tissue and that the fluorescence of the mRFP and cpYFP can be distinguished (Figure 2).

## 4. Discussion

To facilitate the generation of multi-gene constructs, we constructed a bi-directional vector where the expression of two transgenes is driven by back-to-back CaMV35S and Figwart Mosaic Virus (FMV) constitutive promoters. The incorporation of two independent LR-clonase sites in an anti-parallel orientation permitted the integration of two different fluorescent proteins via a single LR-clonase reaction. Dual-integration of independent cDNA into the two clonase sites was observed in 15–30% of screened clones, whereas the remaining clones showed homo-integration of a single cDNA into both sites. Transient transformations of *Nicotiana benthamiana* were carried out to study the functionality of this vector in plant tissue. In both orientations, the FMV and 35S drove constitutive expression of red and yellow fluorescent proteins.

pGEMINI exploits a quirk of the recombination reaction that allows two copies of a target sequence to be introduced into a vector carrying two independent inverted gateway cassettes separated by an intron spacer. This quirk was exploited for the construction of pHELLSGATE [17] and pTKO2 [5], RNAi vectors allowing two copies of a target sequence to be introduced in reverse orientations resulting in a hairpin RNA expression clone. These vectors have been successfully used in RNA interference studies for more than a decade. In pGEMINI, the two inverted recombination sites are separated by back-to-back promoters so that the two independent cDNAs can be introduced from two distinct entry clones in a single reaction. Here we have shown, using fluorescent proteins and transient expression, that this vector allows for the co-expression of two cDNAs in plant tissue, and could be used to facilitate the study of gene-stacking in metabolic processes. In conclusion, the pGEMINI vector represents a unique molecular tool to study the function of a wide array of genes in transgenic plants through transient or stable transformation procedures.
